# Management of Traumatic Cataract with Posterior Capsular Rupture: A Case Report and In Vitro Model Study

**DOI:** 10.1155/2017/4230657

**Published:** 2017-07-18

**Authors:** Wenjuan Wan, Ke Hu, Yan Ji, Can Li

**Affiliations:** The First Affiliated Hospital of Chongqing Medical University, Chongqing Key Laboratory of Ophthalmology and Chongqing Eye Institute, Chongqing 400016, China

## Abstract

**Purpose:**

To investigate the optimal strategy for surgical management of traumatic cataract with posterior capsular rupture.

**Methods:**

We describe four cases of traumatic cataract with posterior capsular rupture and an in vitro model built to evaluate the optimal infusion pressure during surgery.

**Results:**

All patients underwent cataract surgery. By using an anterior chamber maintainer to elevate infusion pressure, we safely performed cataract extraction without phacoemulsification. At 3 days after surgery, visual acuity was greater than 20/25 in all patients, without any complications. Phacoemulsification would also be feasible under anterior chamber maintainer infusion in a similar case of traumatic cataract with posterior capsular rupture during intravitreal injection. In addition, an in vitro model that we established using pig's eyes revealed that the anterior chamber remained stable when the height of infusion bottle was 50–90 mmHg, whereas shallowing of the anterior chamber occurred when the height of infusion bottle was reduced to 40 mmHg, and corneal edema occurred when the height of infusion bottle was raised to 100 mmHg.

**Conclusions:**

During management of traumatic cataract with posterior capsular rupture, using an anterior chamber maintainer to maintain optimal infusion pressure may reduce the risk of anterior hyaloid membrane breakup and vitreous loss.

## 1. Introduction

Traumatic cataract with isolated posterior capsule rupture is a rare complication occurring after blunt ocular trauma [1, 2]. In such cases, the anterior chamber is more unstable during cataract surgeries than it is in cases of anterior capsule rupture because the anterior hyaloid membrane can rupture more easily during the more complicated operative procedure.

Herein, we report four cases of blunt injury-induced cataract with posterior capsule rupture. In all four cases, an anterior chamber maintainer was inserted and optimal infusion pressure was attained before the intraocular procedure was performed. Furthermore, we also describe the establishment of an in vitro porcine model of this condition and provide supporting evidence for our management strategy.

## 2. Case Description and In Vitro Model Results

**Figure 1:**
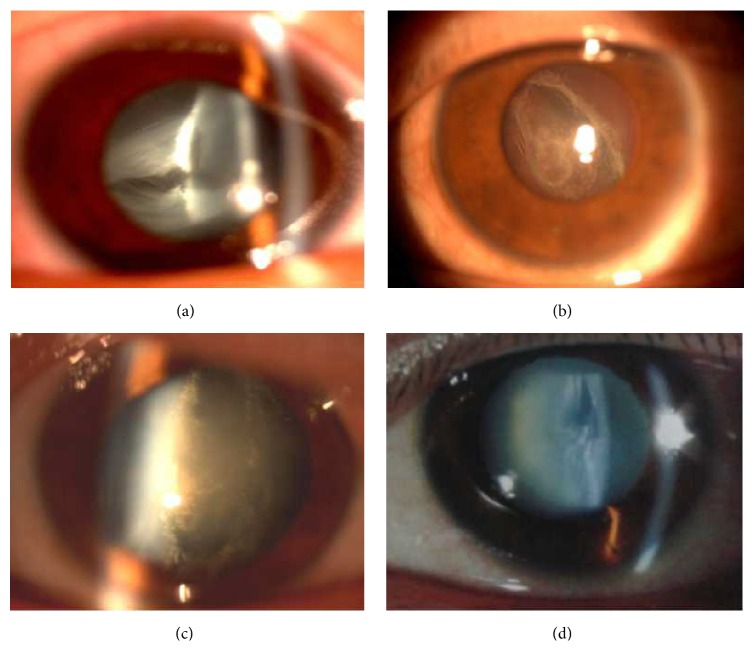
Preoperative slit-lamp photographs of Case  1 (a), Case  2 (b), Case  3 (c), and Case  4 (d). The posterior capsule ruptured transversely (Case  1) or longitudinally (Cases  2–4). A dense lens cortex is visible, especially in the area surrounding the ruptured parts.

All four cases described herein were treated at our department between May 2009 and October 2015. The patients were four men who had experienced blunt eye injuries before the age of 50 years. Of these, only one patient was referred to our clinic soon after experiencing the trauma. In this patient, the sudden vision impairment and blunt pain were mainly caused by corneal edema and hyphaemas. The other three patients visited our clinic more than 2 weeks after their injuries had occurred and presented only with reduced visual acuity. Slit-lamp biomicroscopy revealed normal depth of the anterior chamber, an intact anterior capsule, and a ruptured posterior capsule (transversely in 1 eye and longitudinally in 3 eyes, Figure 1) with or without vitreous prolapse into the opaque lens cortex. Fibrosis of the capsular rupture was observed in all the cases except Case  1 during surgery.

**Figure 2:**
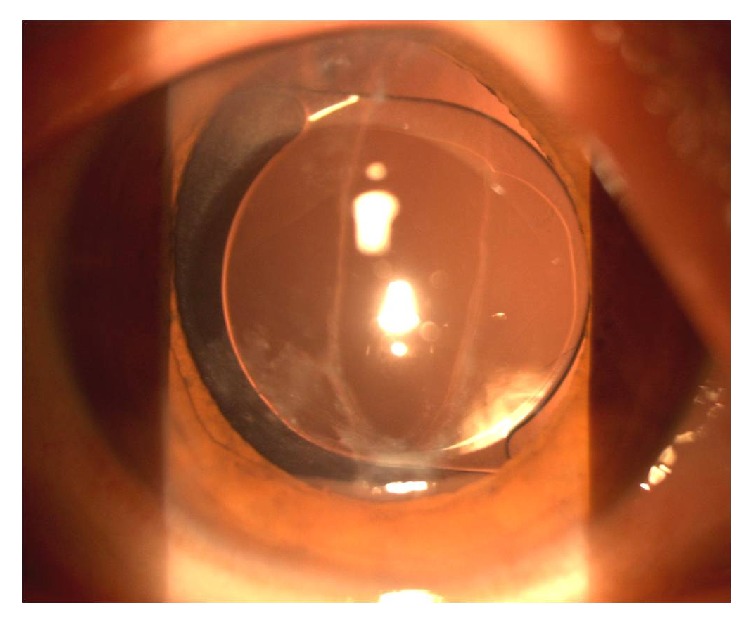
Postoperative slit-lamp photographs of Case  3 with a longitudinally ruptured posterior capsule. The anterior chamber maintainer was placed under lens cortex aspiration, and the intraocular lens was inserted into the bag.

Cataract surgeries were performed when anterior chamber inflammation was well controlled and hyphaema was completely absorbed. A conventional clear corneal cataract incision was created along with two paracenteses, a 20-gauge anterior chamber maintainer was inserted through one of the paracentesis sites, and an infusion of balanced salt solution (BSS) was initiated at the infusion height of 90 mmHg during the surgery. Standard continuous curvilinear capsulorhexis was performed under viscoelastics. Instead of conventional phacoemulsification, the lens nucleus underwent hydrodissection and extracted manually; then an aspiration device was used to remove the cortex through the auxiliary incision. Anterior vitrectomy was performed only in Case  1 to remove the vitreous that had herniated into the posterior capsule tear. A foldable intraocular lens (IOL) was implanted in the sulcus or in the bag (Figure 2). Phacoemulsification through a 1.8 mm incision would also be feasible under anterior chamber maintainer infusion to maintain adequate infusion during the surgical maneuver in a similar case of traumatic cataract with posterior capsular rupture. However the capsular rupture was caused by needle during intravitreal injection.

The uncorrected visual acuity (UCVA) reached 20/25 or better 3 days after surgery, without cells in the anterior chamber, high intraocular pressure (IOP), or any other complications. IOL was stable with no decentration (Table 1).

To help determine whether high infusion pressure helps maintain the stability of the intraocular environment, we simulated a similar condition in in vitro model of porcine eyes in our laboratory. Pig experiments were carried out in accordance with the guidelines on animal care and use of animals in research, which they were approved by the Animal Care and Use Committee of Chongqing Medical University, Chongqing, China. Porcine eyes were obtained from euthanized pigs, and all the procedure proceeded within 2 hours after eyes were enucleated. The posterior capsule was scratched with a 25-gauge needle to induce a vertical tear through the ciliary pars plana. After placement of an anterior chamber maintainer through the paracentesis site, continuous curvilinear capsulorhexis was conducted under viscoelastics. Then, under the infusion of BSS, cataract aspiration was performed. When the height of infusion bottle was reduced to 40 mmHg, the anterior chamber became shallow, the cornea became wrinkled, and the cataract cortex touched the corneal endothelium layer. As long as the height of infusion bottle was maintained between 50 and 90 mmHg, all the procedures could be performed safely without any complications including prolapse of vitreous or drop nucleus (Supplement video in Supplementary Material available online at https://doi.org/10.1155/2017/4230657). When the height of infusion bottle was elevated to 100 mmHg, the anterior chamber was stable but the high IOP induced corneal edema, which resulted in blockade of the operative view. The experiments have been repeated 6 times.

## 3. Discussion

In the present study, favorable prognoses without any complications were achieved in all four traumatic cataract patients with posterior capsular rupture. Because visual acuity might be affected even in the absence of any eye pain, there could be a significant delay in patients seeking treatment after experiencing blunt injuries. In the present cases, we performed cataract aspiration and posterior chamber IOL implantation in the bag, using an anterior chamber maintainer. In only one case, vitrectomy was performed for removal of vitreous that had herniated into the lens capsule. In this case, IOL was implanted in the sulcus because of the horizontally broken posterior capsule and the loss of vitreous.

The anterior chamber maintainer, which facilitates ophthalmological surgical procedures and reduces iatrogenic damage to the iris, corneal endothelium, and lens, has been used in the management of hard nuclear cataract lens [3], subluxation in young patients [4], endoscopic goniotomy [5], traumatic hyphaemas [6], congenital pupillary-iris-lens membrane [7], and spherophakia [8]. As compared to extracapsular cataract extraction (ECCE) with the protection of viscoelastics, anterior chamber might be more unstable when nucleus extraction was performed during phacoemulsification when posterior capsular was ruptured. Anterior chamber maintainer usage might help maintain a steady IOP and anterior hyaloid membrane during surgery, thus reducing the risk of corneal endothelium impairment, vitreous loss, and lens matter dropping into the posterior segment. Furthermore, continuous positive intraoperative perfusion washed out pigments and inflammatory mediators in the anterior chamber, so the postoperative reaction was mild. Because of the abovementioned advantages, anterior chamber maintainers are widely used, including, in our hospital, the management of congenital cataract, congenital deficiency of the posterior capsule, and posterior capsule rupture in cataract surgeries. In a previous study, Li et al. [2] reported a case of traumatic posterior capsular rupture in which the lens matter was removed with a vitreous cutter via the cataract incision, and an anterior chamber maintainer was used only for separate intraocular perfusion. In our cases, the cataract could be aspirated under anterior chamber maintainer infusion, and vitrectomy was only performed when vitreous herniation was present. In cataract surgeries, intraocular pressure is determined by the infusion force (height of the infusion flask), the caliber of the cannulas, and the aspiration force. Since the caliber of the cannulas and the aspiration force were constant to a certain degree, we chose to measure the height of infusion bottle to represent the infusion pressure. Despite the differences between human and pig corneas, our in vitro model demonstrated the importance of maintaining optimal infusion pressure during the surgery.

There are several limitations in our study. The depth of anterior chamber was normal according to slit-lamp biomicroscopy; we assumed that it might be because of the fact that the lens cortex has not been prolapsed out in these four cases; however the exact anterior chamber depth should be determined by ultrasound pachymetry, Pentacam, or IOL master to confirm whether the anterior chamber depth was normal in the future. Furthermore, in our in vitro model, the posterior capsular rupture was created by a 25-gauge needle scratching the posterior capsule. It might not completely represent the condition of posterior capsular rupture caused by blunt ocular injury. And in vivo experiments should be conducted in the future since they would be helpful in determining the time lag between the appearance of capsular rupture and fibrosis development and observing the complications after operation.

On the basis of our experience with the cases described in this report as well as our in vitro model, we recommend the use of anterior chamber maintainer with a stable infusion rate during the surgical treatment of traumatic cataract with posterior capsular rupture only. Maintenance of optimal infusion pressure may reduce the risk of anterior hyaloid membrane breakup and vitreous loss.

## Supplementary Material

In vitro model of posterior capsular rupture was simulated, then lens extraction was performed safely under the perfusion of anterior chamber maintainer, when the height of infusion bottle was 80 mmHg.

## Figures and Tables

**Table 1:** Characteristics of four cases of traumatic cataract with posterior capsular rupture only.

Case	1	2	3	4
Sex	Male	Male	Male	Male
Age (years)	18	33	47	33
Eye	OD	OS	OD	OS
Trauma	Hit by a stick	Impacted by a chair	Struck by a wood	Hit by a stone
Referral time	3 hours after trauma	2 years after trauma	1 year after trauma	15 days after trauma
Operation time	5 days after trauma	2 years after trauma	1 year after trauma	17 days after trauma
Clinical signs				
Cornea and anterior chamber	Edematous cornea, hyphaemas	Not involved	Not involved	Not involved
Lens	Posterior capsule ruptured transversely	Posterior capsule torn longitudinally	Posterior capsule torn longitudinally	Posterior capsule ruptured longitudinally
Vitreous	Vitreous prolapsing into the lens cortex	Not involved	Not involved	Not involved
Surgery				
Phacoemulsification	No	No	No	No
Aspiration	Yes	Yes	Yes	Yes
Anterior vitrectomy	Yes	No	No	No
Anterior hyaloid membrane	Ruptured	Intact	Intact	Intact
IOL implantation	In sulcus	In the bag	In the bag	In the bag
Preoperative UCVA	CF	10/20	HM	HM
UCVA 3 days after operation	20/20	20/25	20/25	20/25
Complications	None	None	None	None

CF: counting fingers; HM: hand motion; IOL: intraocular lens; OD: right eye; OS: left eye; UCVA: uncorrected visual acuity.
